# A Case of Loss of Nose from Syphilis, and Restoration by the Taliacotian Operation; with Remarks

**Published:** 1835-01-01

**Authors:** Frederick Tyrrell

**Affiliations:** Surgeon to St. Thomas's Hospital, &c.


*A Case of Loss of Nose from Syphilis, and Restoration by the Taliacotian Operation; with Remarks.* By Frederick Tyrrell, Esq. Surgeon to St. Thomas's Hospital, &c.

In relating the following case, I shall omit all minute detail, and confine myself to such points as I consider worthy of notice and requisite to elucidate the observations I have subsequently to offer.

J. H. æt. twenty-seven, of small stature, light complexion, dark hair and eyes, and of scrofulous diathesis; by trade a working goldsmith; had suffered from cynanche tonsillaris two or three times, and also from common lepra twice. His habits had been irregular, but he had not been addicted to intemperance.

In 1831 he had gonorrhœa and chancres, for which he took mercury, but not so as to affect his mouth, and he apparently got well. A few weeks afterwards he was attacked with inflammation and ulceration of the soft palate, which destroyed part of the palate and uvula; this disease was arrested and relieved by mercurial fumigations and a gargle of the chloride of soda.

After this he remained tolerably well for about a year; but, in the spring of 1832, he felt uneasiness at the upper part of his nose, which became swollen and tender. This was soon followed by an offensive discharge from the nostrils, which was frequently tinged with blood; and some pieces of bone separated and passed by the pharynx, through the mouth. Under professional advice he took mercury in large and small doses, sarsaparilla, and mineral acids, and applied injections of the solution of the chloride of soda; but, in spite of the means employed, the disease progressed, and in October (six months after the disease commenced in the nose) he applied at St. Thomas's Hospital, and was admitted under my care. At the time of his admission, the extremity of the nose, the fibro-cartilaginous septum, the whole of the left ala, and part of the right, were gone, and the larger part of the turbinated bones had exfoliated; the ulceration was still proceeding, and the edges of the ulcer were ragged, the surface sloughy, and affording a copious thin secretion, mixed with blood, highly offensive and irritating; the surrounding skin was red and swollen, and the whole part excessively tender and painful. He was pallid and emaciated; his pulse was quick and feeble, his appetite bad, his bowels irregular, usually constipated; he enjoyed but little sleep, and frequently woke in profuse perspiration.

I prescribed for him, Hydrarg. c̄. cretâ, g. ij.; Opii, g. j. om.

nocte. Decoct. Sarsap. c. c̄. Quinin. Sulphat. ter die, and a mild aperient when required. His diet was to be nutritious. As a local application I ordered the following lotion, to be used on lint, and covered by a light and warm linseed poultice: ℞. Aquæ Destillatæ, lbj.; Opii, ʒj.; cola post solutionem; tum adde Liquori colato Mucilaginis Acaciæ, ʒj. ut ft. lotio.

For a few days there was no visible improvement, and he obtained very little sleep; I therefore prescribed liquoris opii sedat. min. xxx. to be taken at night, instead of the grain of opium. An improvement then took place in his general health; he rested better, ate with some appetite, and requested to be allowed some port wine, of which I ordered two glasses a day for him. But, although his general health mended, the ulceration did not stop, and the diseased part continued tender, painful, and unhealthy in aspect. About the middle of November his health again retrograded, when I changed his medicines, giving him Ferri Subcarbon. ʒss. ter die, and Solut. Acetatis Morph. m. x. nocte.

His general health again rallied, but the ulcer remained much in the same state; and, in the beginning of December, I tried, as a local application, an ointment of the Hydriodate of Potash; but it created so much irritation, that it was soon discontinued, and a lotion of Black wash, Opium, and Mucilage substituted. During the whole of the period that he had been under my care every attention had been paid to the state of his secretions; and his food, always of nutritious quality, was changed repeatedly, to suit his palate and excite appetite.

The local disease nevertheless extended; the right ala had been destroyed, the ossa nasi had been exposed, and had exfoliated; the vomer and remains of the turbinated bones had also separated. In consequence of some observations in the periodicals on the efficacy of iodine in secondary syphilis, I was induced to try it in this case; and, on the 31st of December, prescribed Solut. Potassæ Hydriodatis (Lugol's) ter die, and the dose was gradually increased, during the month of January, to min. xvj. ter die. He continued the use of the acetate of morphia, of which the dose had been increased by degrees to min. xxx.; and he occasionally took besides a pill of conium and ipecacuanha, to relieve a cough. The exhibition of the iodine was followed with immediate beneficial change: his appetite, in a few days, was excellent; he slept well, and was enabled to leave off his opiate; his cough subsided, and the local disease seemed as if affected by some charm, so rapid was the change in it. The tenderness and pain were no longer felt; the surrounding redness and swelling disappeared; the surface and edges of the sore became even, florid, and healthy, and gave out a good purulent secretion.

From this time he went on well in every respect; his general health improving, and the ulcer granulating and cicatrizing; so that on the 10th of July he was made an out-patient; once only was he obliged to leave off the iodine for a few days, in consequence of his stomach being disordered, and he then took the sarsaparilla again, but resumed the use of the liq. Potassæ Hydriodat. as soon as the irritability of the stomach had subsided.

While attending as an out-patient, the sarsaparilla was substituted for the iodine for the same reason, but his general health declined, and the sore became unhealthy, and began to spread again. I therefore readmitted him into the hospital on the 1st of August; and considering the use of the iodine inadmissible, from the state of the stomach, I directed the sarsaparilla to be continued, with small doses of Hydrarg. c̄. cretâ, and opium, with his former diet.

He was attacked with diarrhœa, which had been, and was prevalent in the hospital, on the 23d of September, which compelled me to omit the sarsaparilla, &c. for about three weeks. On the 15th of October he resumed the use of the sarsaparilla, and was so much recovered on the 19th, that I prescribed two tablespoonfuls of the following mixture, to be taken three times a day, instead of the sarsaparilla: ℞. Iodines, grss.; Potass. Hydriod. ʒss.; Syrupi Papaveris, ℥ss.; Aquæ Destill. lbss. Rapid improvement again took place in his general health and in the ulcer; and, without any change worthy of notice, the ulcer had perfectly healed, and his general health was restored in the beginning of January, 1833.

Some months afterwards he called upon me, to solicit me to try some means of relieving the horrid deformity created by the loss of the parts I have mentioned. I then resolved upon performing the Taliacotian operation for him, there being an ample forehead to furnish integument for the new nose; but, before I undertook the operation, I was desirous that the new formed structures should be well organized, and contracted as much as they would admit. Considering these objects to be effected in the spring of 1834, and his general health being good, I admitted him into St. Thomas's again.

Reflecting on the operation which I was about to undertake, I thought I might, by artificial means, give support and figure to the new nose, which I feared (from the loss of the ossa nasi, and the extensive ravages of the ulceration,) would be otherwise difficult to form and maintain.

The fact of styles or tubes of silver or gold being worn in the nasal duct for years without inconvenience or mischief, principally induced me to make the trial of a metallic support in the present case. I therefore took a cast, in plaster of Paris, of the patient's face, and had a light but firm frame of platina formed, the base of which was to rest on the frontal bone above, and the superior maxillary bone below; from this there projected a small stem, corresponding to the septum of the nose, continuous with, and supporting inferiorly, the main part, which passed in the direction of the median line, from that end of the base which was to rest on the frontal bone, and joined the portion I have mentioned, corresponding to the septum at an acute angle: from near the junction of these two parts, but attached entirely to the dorsal portion, two small alæ projected laterally, which were to support the alæ of the new organ.

The support was made of platina, as not liable to be acted upon by the secretions or air; and in forming it I bore in mind the possibility of its producing irritation or mischief, which might render it necessary to remove it; it was not, therefore, so firm as it might have been made, had I been certain of its remaining without injury, for I had contrived it so that it could be easily removed at any time, without disturbance to any part.

Having fitted the platina frame to the plaster cast I had of the face, I carefully modelled over it some plastic wax, as near as I could judge, of the thickness of the integument of the patient's forehead, so as to form an artificial nose; this being afterwards spread out flat and laid upon the forehead, gave me accurately the portion of integument I should want to cover the platina frame, and form the new nose; but it was necessary further to make allowance for the twisting or turning of the flap, and the contraction which would occur after its union.

Being fully prepared, and the patient being in good health, I performed the operation on the 16th of May, 1834. The extent of integument which was to be raised from the forehead was distinctly marked out by a line in indigo; the stem by which the flap was to retain its connexion was at the inner extremity of the right eyebrow, and the flap itself had an oblique direction over the left eyebrow and left side of the forehead; it terminated in the tongue-like piece which was to form the septum of the nose.

The patient was placed in a recumbent posture, and I first made an incision completely through the integument on the right side of the nasal opening, which extended from the nasal part of the frontal bone, downwards and outwards, to the site of the termination of the original ala; a second incision, parallel to this, but about one tenth of an inch distant, and penetrating to the same depth, included a narrow strip of integument, which I dissected out, leaving a narrow deep groove. I then, in the same way, made a similar groove on the left side.

Next I removed a small square piece of the integument and soft structures on the median line, at the upper part of the commissure of the lip, so as to expose the periosteum over the junction of the superior maxillary bones, to a sufficient extent to receive the corresponding part of the base of the platina support and the extremity of the new frænum. I also made a similar space over the lower part of the frontal bone, on the median line, to lodge the upper part of the base of the platina frame.

Thus I had prepared for the reception of the artificial support, and the skin which was to form the new nose, and I proceeded to detach the flap from the forehead.

I traced the outline, made with indigo, with the scalpel, carrying the point of the instrument to the pericranium; and then I dissected up the flap, leaving little or nothing but pericranium. The connexion left towards the right eyebrow was about a quarter of an inch in width.

The hemorrhage was checked by pressure with German tinder, and the flap was held between sponges dipped in warm water.

As soon as all bleeding of consequence had ceased, I put the platina frame in its place, and brought the flap of integument over it. To secure the flap, I used small curved needles and thin silk ligatures, and passed the first sutures through the extreme lateral parts of the flap, and the lower ends of each lateral groove; and, by tying the ligatures, I readily adapted and fixed the extremity of each ala in its proper situation. I then passed other sutures on each side, about a third of an inch apart, so as to secure the edge of the flap in the groove made for its reception; and lastly, I fixed, in like manner, the strip of skin which formed the septum.

A small vessel near the tongue-like portion of the flap which formed the septum, continued to jet out blood, even a short time after the sutures had all been tied; shewing that a large part of the flap was well organized. Every part fitted perfectly, and I had good reason to be satisfied with the immediate result.

The patient bore the operation without a murmur or complaint; and was conveyed to his ward and bed in high spirits.

the wound on the forehead was covered with German tinder, and the nose had a thin layer of soft cotton placed on it; but the larger part of this covering was soon removed, for the influence of the air from the lungs kept the part perfectly warm.

Union took place very rapidly, and in four or five days every suture had been removed; the adaptation had been so perfect, that it required close inspection to distinguish the line of junction for a considerable extent. The stem of the flap which had been twisted in turning down the flap, had become perfectly smooth. The part retained its sensibility; but if touched, the sensation to the patient was as if the forehead were touched; and this occurred for many weeks after the operation.

For the first two or three weeks everything went on most prosperously: the nose retained an excellent figure, and the platina frame appeared fixed, and did not produce the slightest inconvenience; but I soon observed changes going on, which I dreaded would disturb the harmony of the work. The integument forming the new nose began to contract, and I regretted to see this take place very unequally; that on the left side being much more rapid and extensive than on the right; this I had in a degree anticipated, and thought I had amply provided for it, by making the side of the flap which formed the left nostril more extensive altogether than the other. I had not, however, calculated upon the greater contraction of the thin integument, of which the left side of the nose was formed; for it came from the superior part of the forehead, where the skin was thinner, and the subcutaneous cellular tissue much less abundant.

The irregular contraction after a little time displaced the platina frame, and the part which should have supported the point of the nose, by degrees projected out of the left nostril; still it did not irritate, or occasion any uneasiness.

After waiting some weeks, to ascertain whether I could remedy the defect without removing the platina frame, and deeming it impossible, I reluctantly had it taken out; this was done by the patient himself, easily, and without suffering.

The contraction increased a deformity of the upper lip, which previously existed on the left side, by drawing up and everting that part of the lip. The previous defect had been occasioned by the exfoliation of a portion of the superior maxillary bone, and I am of opinion that the want of support and attachment resulting from this had favoured the contraction of the left ala.

On the 3d of October last I remedied the defect in the upper lip by an operation similar to that for hare lip, and at the same time closed a part of the left nostril, and enlarged the right, by which means the nose was made nearly symmetrical.

At this time the organ appears fixed, and not likely to undergo any further change; it is as good as any that I have seen formed by similar means.

The advantages resulting from the operation have been manifold, and greater than I expected; for not only has the patient got rid of a shocking deformity, but he is now free from a dryness of the nasal cavities, and of the pharynx, which had continually distressed him; and his voice, which was before extremely indistinct and unpleasant, is now clear and perfectly audible.

*Observations.* The history of the case, as far as regards the influence of the syphilitic virus on a patient of weak power and scrofulous diathesis, is by no means uncommon; for although such cases are not so frequent as they were some twenty or thirty years ago, yet our large metropolitan hospitals usually afford examples somewhat analogous. There is scarcely any surgical class of disease in which a greater revolution has taken place in the treatment (within the period I have mentioned) than in syphilitic affections. Mercury, which was formerly considered as a specific in most of these cases, is now confided in by few, is looked upon as a useful auxiliary in the treatment by the many, and is rejected altogether by others. My own experience induces me to consider it as a salutary and useful remedy in very many cases, but certainly not a specific; and I believe many will agree with me when I say that there are cases, as the present one, in which mercury aggravates rather than alleviates the disease. Some such cases as I allude to are relieved by sarsaparilla, and very small doses of mercury; some by sarsaparilla alone; and I have known some do well under means of great variety, but tending to one principal object, viz. the restoration and support of general power.

But in most of these cases the recovery has been very tardy, and often protracted by frequent relapses, and some progressed in spite of all our care, and destroyed life either by exhaustion from continual suffering, or by the extension of the disease to vital organs.

Few will, I think, deny that a more powerful remedy was wanting to enable us to combat the most formidable cases of secondary syphilitic disease with anything like certainty of success.

The influence of the preparations of iodine, as far as present experience goes, will almost warrant us in coming to the conclusion that the much desired remedy has been found. The case I have related was one of the first in which I tried this remedy, and I have but feebly detailed its effect. I have never seen such rapid influence produced upon general and local disease by any other remedy.

I have for the last two years employed it in all the varied forms of secondary syphilitic and of scrofulo-syphilitic disease; in the affections of the throat, of the skin, of the fibrous tissues, &c. I have also given it in many cases of primary disease, and with excellent effect, when the local action has been unusually disordered.

Further experience is of course necessary to enable us to form very accurate opinions respecting the use of such a remedy; but I shall briefly state my opinions of it, resulting from the experience I have had. I have found its effects most rapid in secondary syphilis, modified by scrofula, when the general power has been depressed, and a morbid irritability has existed.

In all cases where it acts beneficially it produces rapid effects on the general health; it increases appetite, augments the force of the circulation; it lessens nervous irritability, and subdues local pains.

Almost as soon as its influence is apparent in the system, the local affections undergo salutary changes, without the aid of other than the most simple applications.

There are circumstances, however, under which its exhibition must be delayed or laid aside; as when the stomach is very irritable; when there is a tendency to or an actual state of diarrhœa; again, when the secretions from the alimentary canal are ill-conditioned, or when there is constipation.

The form I prefer is that which I have described as the Mistura Potassæ Hydriodatis. Sometimes I omit the poppy syrup, and sometimes I omit the iodine, giving the hydriodate of potash simply. These alterations are determined by circumstances afforded by respective cases; thus, if there be no nervous irritability, if there be headach, or tendency to confined bowels, I omit the poppy; if the stomach is naturally irritable, I omit the iodine, &c.

I have hitherto found the small dose I have mentioned quite sufficient; and in some cases have been obliged to diminish it, as it created nausea.

The Mistura Potassæ Hydriodatis does not disorder the stomach so soon or so frequently as Lugol's solution, or other somewhat similar formulæ that I have tried.

In many cases I have found much benefit from giving at the same time small doses of mercury, as a grain or two of the Hydrarg. cum Cretâ or Blue Pill at night, or every second night, to promote a proper degree of biliary secretion.

Like all other remedies, it must be given at proper times, and under circumstances favourable to its use. I am satisfied of its being a very valuable remedy, and hope that an indiscriminate and improper use of it will not bring discredit upon it. When I have had more experience I may have opportunity to offer some farther observations on the use of this remedy, in a more connected and extended form than I am at present able to do.

I shall in conclusion offer a few remarks on the operation and its effects.

With the exception of the introduction of an artificial support, I believe there was nothing novel in the plan I pursued, but it may nevertheless be useful to detail the principal difficulties.

In calculating the extent of the flap of integument to be raised from the forehead, full allowance must be made for the turning and for the after-contraction. If the flap is to remain connected to the right side, in turning it down the sides become reversed; that which had been to the right being placed afterwards to the left, and the left to the right. Supposing them to be of equal length, after being turned down, that on the right side would be relaxed, and that on the left be stretched out; to obviate this, the left side of the flap should be made considerably longer than the right; if the integument which is to form the flap varies much in thickness, a further allowance should be made, as the thinnest portion will afterwards contract most.

In another case, if I made the septum with the flap, I should secure it first, as I experienced some difficulty in doing so after I had fixed the flap itself. If, however, the forehead was not sufficiently large to afford integument for the septum, without encroaching on the hair, I should form the septum subsequently from the upper lip, as recommended by Dieffenbach.

Short curved stiff needles enabled me to pass the sutures with great ease; and I much prefer the small silk or thread ligatures to the metallic thread, or metallic pins. I have used the last two in other operations, but cannot detect any advantages in their use.

The experiment of the platina frame as a support failed only, I consider, from peculiarities in the case, and from my having given it a figure which made its displacement easy. I am perfectly satisfied that such a support might be used with good effect, and without its producing any mischief; the want of such aid renders the new-formed organ usually irregular and misshapen; this has been the case in every instance which I have seen.

Before the parts are adapted and fixed by sutures, the surgeon should wait until all hemorrhage which may be likely to interfere with the surfaces has ceased, otherwise coagula may be deposited, which would prevent adhesion.

In the after-treatment the patient should be kept recumbent, and at perfect rest, and should be desired to suppress an inclination to cough or sneeze. There is no fear of the part suffering from want of warmth; the warm air from the lungs keeps up sufficient heat. I think it not improbable that the part might be so heated and distended as to require the influence of cold, and the erect posture to check mischief. I should, however, not consider it prudent to apply cold without evidence of its necessity.

As soon as the union is firm, the sutures should be removed, to prevent their irritating and exciting ulceration.

The loss of the nose not only produces conspicuous deformity, but subjects the patient to consequences which materially affect his comfort and health. The constant dryness of the nasal cavities and of the pharynx, with frequent soreness of the latter, disturb the rest, and affect the appetite; and the effect on his articulation is such as to render it extremely indistinct and unpleasant. He is, by his misfortunes, almost an outcast from society.

Are we not then warranted in urging the performance of an operation by which most of these evils can be remedied—which is not attended with risk, and which, if carefully conducted, can hardly fail? Such is my impression, from the result of the present case, and others which I watched.

17, *New Bridge Street, Blackfriars.*
*Dec.* 27, 1834.

